# Urine Treatment on the International Space Station: Current Practice and Novel Approaches

**DOI:** 10.3390/membranes10110327

**Published:** 2020-11-02

**Authors:** Federico Volpin, Umakant Badeti, Chen Wang, Jiaxi Jiang, Jörg Vogel, Stefano Freguia, Dena Fam, Jaeweon Cho, Sherub Phuntsho, Ho Kyong Shon

**Affiliations:** 1 School of Civil and Environmental Engineering, University of Technology, Sydney (UTS), City Campus, Broadway, NSW 2007, Australia; Federico.Volpin@student.uts.edu.au (F.V.); Umakant.Badeti@student.uts.edu.au (U.B.); Chen.Wang-7@student.uts.edu.au (C.W.); Jiaxi.Jiang@student.uts.edu.au (J.J.); Sherub.Phuntsho@uts.edu.au (S.P.); 2 Aquaporin A/S and Aquaporin Space Alliance (ASA), 2800 Kgs. Lyngby, Denmark; jvo@aquaporin.dk; 3 Department of Chemical Engineering, The University of Melbourne, Victoria 3010, Australia; stefano.freguia@unimelb.edu.au; 4 Institute of Sustainable Futures, University of Technology, Sydney (UTS), City Campus, Broadway, NSW 2007, Australia; Dena.Fam@uts.edu.au; 5 School of Urban and Environmental Engineering, Ulsan Institute of Science and Technology (UNIST), UNIST-gil 50, Ulsan 689-798, Korea; jaeweoncho@unist.ac.kr

**Keywords:** human urine, resource recovery, international space station, urine fertiliser

## Abstract

A reliable, robust, and resilient water recovery system is of paramount importance on board the International Space Station (ISS). Such a system must be able to treat all sources of water, thereby reducing resupply costs and allowing for longer-term space missions. As such, technologies able to dewater urine in microgravity have been investigated by different space agencies. However, despite over 50 years of research and advancements on water extraction from human urine, the Urine Processing Assembly (UPA) and the Water Processor Assembly (WPA) now operating on the ISS still achieve suboptimal water recovery rates and require periodic consumables resupply. Additionally, urine brine from the treatment is collected for disposal and not yet reused. These factors, combined with the need for a life support system capable of tolerating even dormant periods of up to one year, make the research in this field ever more critical. As such, in the last decade, extensive research was conducted on the adaptation of existing or emerging technologies for the ISS context. In virtue of having a strong chemical resistance, small footprint, tuneable selectivity and versatility, novel membrane-based processes have been in focus for treating human urine. Their hybridisation with thermal and biological processes as well as the combination with new nanomaterials have been particularly investigated. This article critically reviews the UPA and WPA processes currently in operation on the ISS, summarising the research directions and needs, highlighted by major space agencies, necessary for allowing life support for missions outside the Low Earth Orbit (LEO). Additionally, it reviews the technologies recently proposed to improve the performance of the system as well as new concepts to allow for the valorisation of the nutrients in urine or the brine after urine dewatering.

## 1. Introduction

While separate collection, treatment and reuse of human urine is increasingly adopted here on Earth, on the International Space Station (ISS) it has always been a priority. That is because, despite the current technological advances in space transportation, the cost of shipment in space remains over $10,000 per kg [1,2]. This is a strong incentive to reduce the payload mass which, in a best-case scenario, amounts to 15 kg/day considering life support consumables such as air, food and water only [1]. However, recycling water and waste produced on the ISS can reduce this value to less than 1 kg/day [3,4]. As such, urine recovery and reuse is of maximum priority to meet the crew’s demand for drinking and hygiene water, as well as for on-site oxygen generation [5]. Therefore, Exploration Life Support (ELS) or Bioregenerative Life Support Systems (BLSS) are increasingly investigated for on-site regeneration of water, oxygen and nutrients from urine [3,6,7]. These are systems designed to support the biological needs of crew members on board the ISS. The ideal life support systems in space must provide the highest possible percentage of water recovery with the minimum operation and maintenance costs. System reliability and the need for virtually no supply or maintenance is especially important in space. This is especially true for future permanent habitation in life support systems. On the other hand, the initial capital cost requirements, necessary for an ELS/BLSS to perform reliable high water recovery and high permeate quality, are more relaxed [8].

In this context, it is no surprise that space agencies such as NASA and ESA have been spearheading the research and development of technologies to reuse human waste streams from crew members for the last five decades [9,10]. Their work on the understanding of urine’s chemical composition and concentrative properties has paved the path for the current research in this field [11]. The drivers behind this work were that, in the context of bioregenerative life-support in space, recovering water and nutrients from urine is necessary to meet the required water/food demand for missions outside Low Earth Orbit (LEO) [12]. As a beneficial side effect, on-site urine processing also allows the large amount of human waste produced during the mission to be better dealt with. It was calculated that, on a 30-month-long mission, each crew member would require 2250 kg of water and 1359 kg of food while producing 1493 kg of urine [13]. Given that water makes up about 95% of urine composition, its dewatering could theoretically supply over 60% of the crew’s water demand, while the remaining brine could be used as plant growing media. In fact, the absence of bacteria/viruses and high nitrogen-phosphorous-potassium (N-P-K) concentrations in urine makes it an ideal raw material for fertiliser production [14]. As such, the transformation of urine brine into a safe fertiliser could be integrated into the hydroponic food production system on board the ISS, where feasibility is already proven through the “Veggie” project [9,15].

As such, this article aims at reviewing the state-of-the-art technologies for urine processing on the ISS and current challenges and opportunities for new alternatives in human waste management. The review especially focuses on membrane-based approaches given their versatility, tuneable selectivity, light weight and chemical resistance. Ultimately, the objective is to outline the current needs to achieve a urine processing unit which is reliable and efficient.

## 2. The Dynamic Urine Composition 

**Figure 1:**
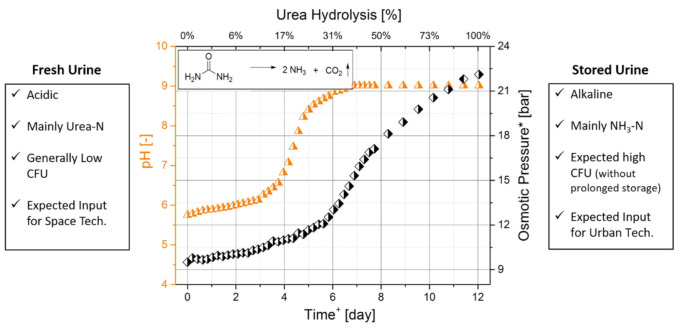
From fresh to hydrolysed. The change in pH and osmotic pressure during urea hydrolysis in the urine. * OLI Studio Analyser software was used to estimate the osmotic pressure based on the chemical analysis of the urine solution. Adapted from Volpin (2019) [19].

Understanding the change in the chemical and biological composition of urine is crucial in selecting the most appropriate urine treatment technology. The storage of urine in non-sterile conditions or at elevated temperatures (>40 °C) causes the urea to hydrolyse into NH_3_/NH_4_^+^ and CO_2_ leading to an increase in the pH, conductivity and osmotic pressure [16,17,18]. Figure 1 shows how the hydrolysis of urea affects the chemical composition of urine. This graph was obtained by continuously measuring the pH, electric conductivity, anions, cations, NH_3_ and urea concentration of a urine sample exposed to environmental conditions for 12 days [19]. This led to the distinction between urine pre-urea and post-urea hydrolysis. The former is generally referred to as “fresh urine” while the latter as “hydrolysed urine”.

Table 1 and Table 2 show the average composition of fresh and hydrolysed urine. Although the actual values of the compounds in the urine vary depending on diet and environmental conditions as well as physical exertion, we excrete, on average, approximately 1270 g_urine_ p^−1^ d^−1^ [14,20]. This accounts for less than 1% of the overall wastewater volume. Nitrogen in the form of urea/NH_3_ is the major constituent (5–10 g/L), followed by K^+^ and Na^+^ (1–2 g/L) [21,22]. Table 2 also shows the high occurrence and concentration of pharmaceuticals, hormones and antibiotics in the urine [23,24]. The concentrated nature of human urine causes the concentration of xenobiotics to reach values even in the ppm range [24].

### Urine in Space

The urine collected in space from crew members generally presents higher calcium concentrations compared to the same urine if collected on Earth. This is because of the renal adaptation and compensation induced by the change in bone stress during microgravity [29,30,31]. In microgravity, the combined effect of reduced fluid intake, hypercalciuria and the presence of nanobacteria all contribute to increased kidney stone formation [32]. As a result, during long-term microgravity exposure (>1000 days) the calcium metabolism is altered with a decreased intestinal calcium absorption and increased urinal calcium excretion [29,33]. In addition, the reduced urine output observed during space missions is likely to magnify the saturation of solutes like uric acids, cystine, struvite, calcium oxalate and calcium phosphate [32]. This causes both a physiological issue (as it can cause diseases like kidney stones) and technical problem as high calcium levels can cause CaCO_3_, Ca_3_(PO_4_)_2_ and CaC_2_O_4_ scaling on pipes or appliances used on the ISS. After studying the urine biochemistry of 332 astronauts, pre- and post-flight, after landing, an 18% and 9.8% increase in hypercalciuria and hypomagnesuria and lower urinary pH were measured [32]. To reduce the bone mass loss problem, during space flights dietary calcium intake of 800 mg/day was also recommended. Overall, the change in the concentration and ratio of monovalent and multivalent cations in the presence of microgravity poses significant problems to the onsite treatment of urine as it promotes scaling and clogging of the equipment.

## 3. Current Practice on the ISS

**Figure 2:**
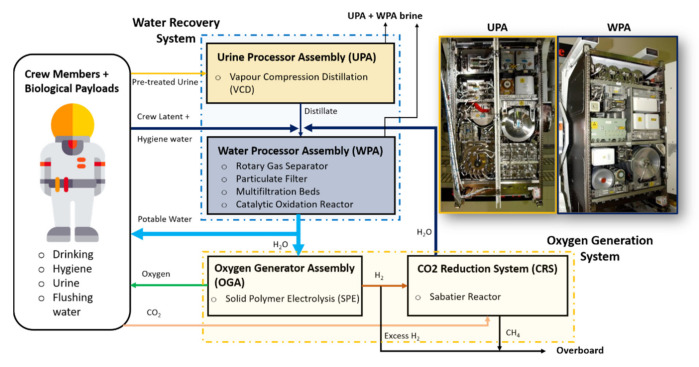
Schematic of the water recovery and management architecture for the US International Space Station (ISS) segment. Adopted and modified from [5,34].

The Water Recovery and Management (WRM) system implemented on the ISS ensures availability of potable water for crew drinking and hygiene, oxygen production and urinal flush water [5,12,34]. The primary sources of wastewater that can be reclaimed and reused on long-term space missions are hygiene, urine and crew latent wastewater, where the last one is the condensed water vapor from crew perspiration and respiration [35]. Figure 2 shows the schematic diagram of WRM currently implemented on the ISS [5,34]. Besides the wastewater produced by the crew, the WRM receives the water produced from the carbon dioxide reduction system, which uses the Sabatier technology to produce water from CO_2_ and H_2_ [5,34]. The WRM processes the received wastewaters (i.e., crew urine, humidity condensate and Sabatier product water) to potable standards [34]. The water recovery systems can be subdivided into two sections:

1. Urine Processor Assembly (UPA) and,
2. Water Processor Assembly (WPA).

UPA only treats urine, while WPA treats the humidity condensate, hygiene wastewater, Sabatier product water and the water treated by the UPA. The details of the UPA and WPA are discussed below.

### 3.1. Urine Processor Assembly (UPA)

**Figure 3:**
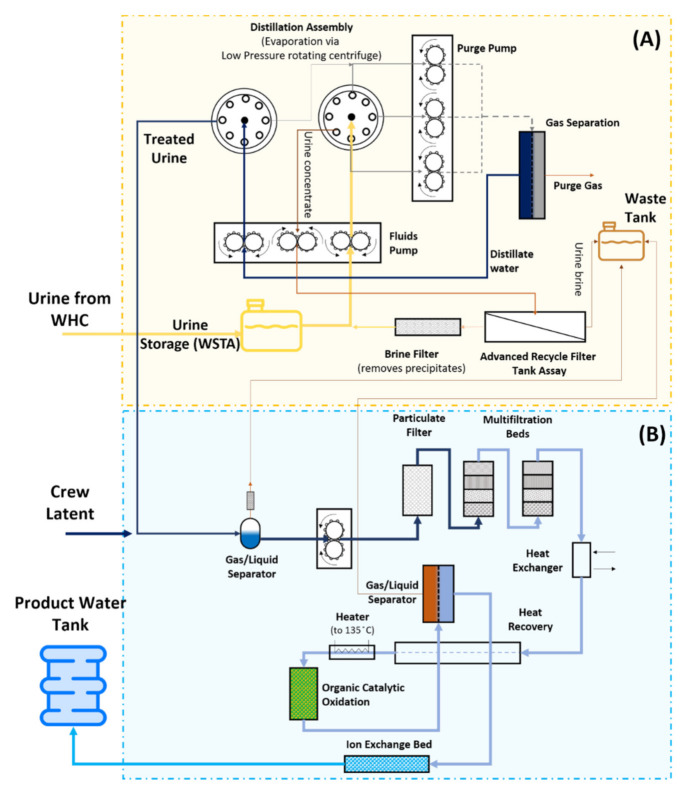
Schematic of the Urine Processor Assembly (UPA) (**A**) and the Water Processor Assembly (WPA) (**B**) installed at the ISS. Adapted and modified from [5,36].

Between 2008 (initial UPA operation) and 2017, the total UPA distilled water production was over 11.2 m^3^ [5]. This produced water from the UPA is sent through the WPA unit for further purification before being used for potable purposes and oxygen generation (see Figure 3). Initially, to prevent microbial growth and chemical stability, urine is stabilised using a mixture of H_3_PO_4_ and Cr^6+^ and stored in the Wastewater Storage Tank Assembly (WSTA) until reaching a sufficient quantity for the treatment unit to start [12]. Afterwards, the stored urine is then pumped to the distillation assembly (DA) (Figure 3). The distillate stream will then go through a gas separation unit, which separates the water from the purge gas, and back to the DA before it can go to the WPA. 

The DA unit, in particular, uses a low-pressure rotating vapour-compression evaporation system to promote the urine distillation process [5,34]. Finally, a firmware controller assembly provides the online monitoring, command control and data downlink for UPA sensors and effectors [34]. The UPA was designed for a nominal load of 9 kg/day and water recovery of 85%. However, up until 2016, it operated at water recoveries of only 70–75% due to CaSO_4_ precipitations which caused severe hardware scaling [34]. To cope with the higher Ca^2+^ concentration in the crew’s urine, a new type of pre-treatment, using H_3_PO_4_ instead of H_2_SO_4_, was adopted which allowed the UPA to return to 85% recovery on ISS [36]. 

### 3.2. Water Processor Assembly (WPA)

The distilled water produced with the UPA is then sent to the WPA unit (Figure 3). There, it is combined with the condensate from the temperature and humidity control system and the Sabatier product water, degassed and sent through a particulate filter followed by multifiltration beds, where inorganic and non-volatile organic compounds are removed [5,34,37]. Following the multifiltration beds, the low molecular weight organics in the wastewater are oxidised in the catalytic reactor that, in the presence of gaseous oxygen feed and 130 °C, has a conversion efficiency of over 95% [38]. After purging the remaining oxygen and gaseous by-products via liquid-gas separation, the carbonate and bicarbonate ions produced during the oxidation are removed via an ion-exchange bed [38]. The ion-exchange bed also provides iodine (or silver) for residual microbial control [36]. Finally, regenerative heat exchanges recover the heat from the catalytic oxidation. The total organic carbon (TOC) concentration of the product water can be evaluated on board the ISS to check if the filters need to be replaced [36,39]. So far, there have been six instances where the TOC analyser on board the ISS detected a spike in the TOC concentration. In such cases, if the concentration exceeds the potable specification of 3000 µg/L, both multifiltration beds are replaced. Additional analysis of water samples is conducted on the ground.

### 3.3. Major Challenges of the Current WRM System

The ISS water recovery system has been operating since November 2008, producing over 30,000 L of water during that time. In this period, on average, 0.21 kg of disposable hardware was consumed for every 1 L of water produced [12]. 

The primary challenges with the design and operation during the first ten years were mainly related to the implementation of the technologies described in Figure 3 under a microgravity environment. The absence of gravity was found to alter the two-phase fluid dynamics as well as increasing the impact of particulates on the system performances. Additionally, the system’s maximum water recovery is strongly affected by the high calcium content in the crew’s urine, which was found to cause CaSO_4_ precipitates on the distillation assembly of the UPA [40]. The distillation assembly is also affected by the urine pre-treatment method. The current use of strong inorganic acids to lower the pH of urine is causing corrosion in the distillation unit [5]. However, changes in the urine pre-treatment solution should be carefully designed not to increase microbial growth. That happened in 2010 when the biomass that formed on the wall of the wastewater tank detached and caused clogging of a solenoid valve, causing it to seize [41]. The multifiltration beds were also found to be unable to remove dimethylsilanediol (DMSD) from the wastewater. DMSD originates from the volatile methyl siloxane (VMS) compounds in the ISS cabin atmosphere [42,43]. DMSD is initially removed by the anion exchange resin in the multifiltration beds and partially removed by the WPA catalytic reactor. Once the ion-exchange resins are saturated, the catalytic reactor is unable to entirely remove it. As such, DMSD is causing a faster replacement rate of the multifiltration beds, doubling the expected mass turnover of the system [44]. 

An alternative to overcome the current wastewater treatment challenges and make use of current waste products are described more in detail in the next section.

## 4. New Alternatives for Urine Treatment in Space

The present WRM closed-loop life support system installed at the ISS is still not optimal due to limitations in water recovery, resupply cost and maintenance [41]. An alternative to the current UPA system must also be highly reliable, durable, lightweight and capable of high recoveries [41]. Reliability is especially necessary for deep space exploration, as the replacement of broken parts is not possible. On the other hand, the capital costs of the process (CAPEX) can be more flexible compared to the necessary low operational costs (OPEX). Currently, essential upgrades for the WRM at the ISS would be:

To improve the current stabilisation and disinfection of urine wastewater to ensure higher water recoveries and long-term microbial control [12].

➢ To increase the water recovery rates beyond 85% and potentially broaden the types of wastewaters that can be processed.
➢ To reduce the reliance on expendable media (e.g., filtration media, sorbents and ion-exchange resins) to minimise the resupply cost and maximise the use [12,37].
➢ To transform the produced wastes (e.g., brine from the UPA and WPA) into edible and valuable produce [2].
➢ To increase the reliability and redundancy of the system while reducing its maintenance and operational cost [2,5,12].
➢ To improve the dormancy of the system. Dormancy refers to the capability to maintain functions following lengthy downtime periods, typically to support crew activity for at least one year, followed by a dormant period of up to one year, and subsequently for an additional crewed mission of up to one year [45].

In the sections below, new emerging technologies in the treatment and reuse of urine and the wastewater produced in space are described.

### 4.1. Urine Stabilisation and Disinfection

Urine stabilisation is crucial in space as urine is very prone to serving as a substrate for bacterial growth, which would cause carbonates and phosphates scaling in the WSTA tanks, pipes and other treatment apparatuses. Currently, urine is mixed with flushing water and stabilised using a pre-treatment formula containing chromium trioxide and sulphuric acid to control microbial growth and urea hydrolysis [5,34,46]. However, this pre-treatment is inherently hazardous, it needs to be resupplied, and it limits the recovery via distillation to <75% [47]. By changing the acid from sulphuric to phosphoric, an extra 10% water recovery was achieved, which allowed for a net saving of over 200 kg of water resupply each year. Together, payload savings and increased water recovery rates also reduced the volume of brine produced each year. Currently, NASA is analysing the brine returned to the ground to evaluate options to increase water recovery above 85% [39,48].

One of the “Green Pre-Treat” solutions proposed to mitigate the side effects of toxic chemicals is to substitute mineral acids (i.e., H_3_PO_4_, H_2_SO_4_) with organic acids (i.e., citric, benzoic) and replace Cr^6+^ with other inhibitory compounds such as quaternary amines [12,47]. An alternative way to reducing the pH of urine and converting urea into stable NH_4_^+^ and NO_3_^−^ is using biological nitrification [3,6,49]. The advantages of biological oxidation of urine wastewater are the prevention of the NH_3_ (g) loss during distillation, reduction of volatile organic carbon (VOC) load to the adsorptive beds with finite capacity and reduction in downstream growth potential [46,49,50,51]. Jackson et al. (2017) [46] showed that oxidising urine biologically, with the addition of organic acids (i.e., 1 g/L benzoic acid or 5 g/L citric acid), could be an alternative to the current storage method. The complexity of an additional biological processor to produce a low pH solution would need to be benchmarked with the current system. 

New advanced oxidation processes (AOPs) have also been proposed for the treatment of human urine in space. Non-thermal plasma (NTP) is one example [52,53]. NTP uses electric discharge at the water-gas interface to generate multiple reactive species, such as OH^•^, O_3_, H_2_O_2_, which are able to mineralise the residual VOCs [54]. This AOP process discharges high energy electrons at the gas-liquid interface which produce highly oxidative species like hydroxyl radicals, able to mineralise the pollutants without the risk of harmful by-product formation [55]. As such, it has been recently used to degrade refractory or challenging compounds such as phenol, crystal violet, methylene blue, acid red 88 and pentoxifylline [54,56,57,58] as well as effectively inactivating faecal coliforms and *E. coli* [59,60]. When used on recalcitrant phenolic compounds, different NTP configurations showed removal rates up to 100% with energy consumption ranging from 0.12–13 g kWh^−1^ [53]. At present, only one article has been published on the use of NTP on human urine for space application [52]. 

To conclude, the development of safe biocides that would ensure long-term antimicrobial effectiveness has been identified as a necessary field of research [12].

### 4.2. Membrane-Based Urine’s Water Recovery Systems

The use of hydrophilic or hydrophobic membranes for wastewater treatment in microgravity is not a new concept. Cath et al. (2005) [61] investigated the possibility of direct osmotic concentration via forward/reverse osmosis (FO/RO), osmotic distillation (OD) and membrane distillation (MD). 

#### 4.2.1. Hybrid forward Osmosis—Reverse Osmosis

Cath et al. (2005) [61] initial concept was to use FO as pre-treatment for RO to reduce the organic and inorganic fouling on the RO membrane. In this process, water is drawn, across the selective FO membrane, from a low concentration wastewater solution to a higher concentration draw solution (DS). The excess water in the diluted DS is then recovered via RO. The double barrier provided by the FO and RO membrane would also ensure a high rejection of organics and pollutants. The rationale behind the choice of FO pre-treatment was the relative lower fouling propensity, low energy consumption, simplicity and reliability [35]. Siddiqui et al. recently showed that FO has a higher fouling propensity compared to RO, but at the same time, it offers superior flux stability against fouling [62]. Conventional hydrophilic polymeric FO and RO membranes were not able to achieve sufficient rejection of small and uncharged compounds, such as urea, as well as volatile organic compounds.

#### 4.2.2. Hybrid forward Osmosis—Osmotic Distillation/Membrane Distillation

To obviate the low rejection of urea, OD or MD were proposed as a DS recovery method. The OD/MD membranes are hydrophobic, meaning that only volatile compounds can pass through. Since urea is non-volatile, OD/MD membranes would theoretically have a nearly perfect urea rejection [35,61]. 

Both MD and OD are membrane distillation processes in which the driving force for the separation is the partial vapour pressure difference across a microporous hydrophobic membrane. While OD is an isothermal process, in which the partial vapour pressure difference is induced only by the salinity concentration difference, in MD, temperature differences between feed and permeate and/or vacuum (VMD) are used to enhance the water transport [63,64]. Because of the very low water flux measured (<1 L m^−2^ h^−1^), OD has not yet proven to be technically and economically feasible [35,61,65,66]. The average water flux of commercial MD and VMD membranes, on the other hand, are in the range of 10–40 L m^−2^ h^−1^ (depending on the temperature difference and applied vacuum pressure) [67,68,69]. More recently, Liu et al. (2016) [64] tested the effectiveness of commercial FO and MD membranes in water extraction from real urine. In this paper, it was shown that if FO does not have a near-complete rejection of urea and NH_3_, the accumulation of those compounds in the DS would cause an NH_3_ permeation in the MD-produced water. Additionally, MD operation temperatures higher than 40 °C are not recommended as the urea in the DS would thermally decompose to NH_3_, which is volatile and can pass through the MD membrane [18]. New FO or RO membranes should be able to achieve near-perfect rejection of urea to ensure acceptable NH_3_ and TOC levels in the permeate water. This is not yet possible with conventional polyamide- or cellulose triacetate-based membrane.

### 4.3. Emerging New Materials to Enhance Water Treatment Efficiency

The successes in the fabrication of membranes with the incorporation of novel materials such as aquaporin vesicles and graphene oxide (GO), has sparked new interest in the application of these new membranes for the treatment of astronauts’ wastewater [41,70,71]. Aquaporin-based membranes, for example, have already been tested on board the ISS by NASA in collaboration with the Danish Aerospace Company (ApS) and Aquaporin Space Alliance (ASA) [70]. This is explained more in detail in the section below.

#### 4.3.1. Aquaporin-Based Membranes

Aquaporins are a class of transmembrane proteins, found in almost every organism and represented in every major taxonomic group, and act as perm-selective water channels [72,73,74,75]. Once stabilised in a polymeric vesicle they can be embedded in the active layer of conventional thin-film-composite polyamide membranes to enhance their water permeability, salt rejection and organic compounds rejection [72,76]. Because of their high rejection capabilities, Aquaporin Inside^®^ hollow fibre (AqP HF) membranes, used in forward osmosis configuration and commercialised by Aquaporin A/S, were tested on Earth and at the ISS as a possible candidate to replace the current multifiltration beds [70,71]. Initially, NASA tested the TOC and DMSD rejection of Aquaporin Inside^®^ membranes using a synthetic ISS condensate feed solution developed by Marshall Space Flight Centre (MSFC). A hydraulic pressure of 20 psi was applied. The results showed a rejection of 61.0 ± 2.8% and 93.3 ± 0.4% of TOC and DMSD, respectively [76]. These rejections were higher than the targeted 50% rejection. As a result, the NASA Advanced Exploration System (AES) initiated a program to evaluate the performance of the AqP HF on the ISS. The program aimed at verifying the effect of real wastewater and microgravity on membrane flux and rejection.

Previous studies of FO membranes had shown reduced flux rates in microgravity due to concentration polarisation (CP) build up on the membrane surface. This was attributed to the absence of buoyancy-driven mixing in space, where all mixing occurs due to Brownian motion [76,77]. Enhanced CP can also affect contaminant rejection as it increases the concentration difference on the surface of the membrane. The water flux results showed a flux ranging from 0.7–2.3 L/m^−2^ h^−1^, which is similar to the average flux of 0.76 L m^−2^ h^−1^ achieved in on-ground tests. Therefore, the effect of microgravity on flux seems to be less severe than expected. Unfortunately, the concentration of TOC and DMSD measured on Earth are questionable as the samples were heavily delayed and not properly stored. These tests will be repeated in a future flight test for validation [76].

#### 4.3.2. Graphene Oxide-Based Adsorbents/Membranes

In a recent paper by Buelke et al., GO-based RO membranes were proposed due to their stability, non-toxicity and high ion rejection capabilities [41]. The authors suggested that GO could enhance the UPA and WPA treatment process if used as (1) adsorbent, (2) membrane or (3) coating solution.

If used as an adsorbent, it could replace the activated carbon in the multifiltration bed due to its higher adsorption capacity [41,78,79]. Additionally, GO could be a potentially better candidate to sequestrate DMSD because of its similar size and charge to Eosin Y, which is adsorbed at a capacity of 300 mg/g by GO [41].

When GO is incorporated in the active layer of a semi-permeable membrane, this could be operated in RO mode (i.e., hydraulic pressure is applied to overcome the osmotic pressure of the wastewater) to separate the contaminants from pure water physically. It was suggested that this could potentially make the distillation assembly, particulate filter and gas separator unit unnecessary. However, this would imply that, firstly, the system should reach hydraulic pressures higher than the osmotic pressure of the concentrated wastewater, at 85% water recovery, which, itself, is a challenge in the absence of gravity. Secondly, there is still a lack of data on the urea rejection proprieties of GO membranes.

Finally, Buelke et al. (2018) [41] proposed using GO antimicrobial proprieties to mitigate the effect of the biomass growth issue that affects the WRM. GO coating could be used as a passive solution to reduce biofilm formation. This, however, would not affect the root cause of microbial growth in the wastewater.

## 5. Nutrient Recovery and Valorisation Projects

Currently, there is no recovery and reuse of nutrients like nitrogen and phosphorous on the ISS. Urine brine (after the UPA) and faecal material are just collected and returned to Earth [3]. However, the breakdown and transformation of wastes into building blocks for edible biomass production is essential to sustain human life during isolated long-term missions [3]. As such, since 50–64% of the nitrogen waste on the ISS comes from crew urine (i.e., 7–16 g_N_ d^−1^), its reuse as a nutrient source will be necessary [3,80,81]. If transformed into a safe and effective fertiliser, urine could be used to grow plants hydroponically on the ISS and on board other vessels or habitats. This concept is even more realistic after the success of the “Veggie” project (or Vegetable Production System) which saw the successful study of plant growth in microgravity [15]. In fact, in April 2014 the SpaceX Dragon capsule sent a low mass, power and maintenance growth chamber to the ISS. The Veggie system hardware, comprising of LED lighting, a fan and a cooling system, was installed and tested growing *Lactuca sativa (VEG-01)*, *Brassica rapa var. nipposinica and Brassica rapa var. chinensis (VEG-03)* [15]. Following the success of the Veggie system, a new growth chamber was then sent to the ISS, called Advanced Plant Habitat (APH). The APH was enclosed and automated, with cameras and over 180 sensors to monitor plant growth. By controlling the moisture, water recovery, temperature and minimising the outside intervention, APH yielded great results when growing *Arabidopsis thaliana* [82].

### 5.1. MELiSSA PROJECT

**Figure 4:**
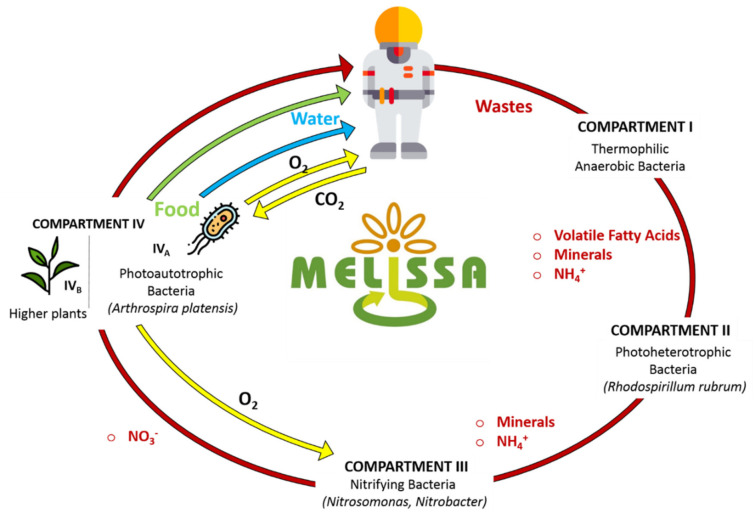
The Micro-Ecological Life Support System Alternative (MELiSSA) loop. Adapted from [84].

In this context, in 1987 the European Space Agency (ESA) envisioned a project to create a Bioregenerative Life Support System (BLSS) to be deployed on the ISS. That was the beginning of interdisciplinary collaboration projects such as the Micro-Ecological Life Support System Alternative (MELiSSA). The core idea was to develop a biological system to close the waste to resources loop. Specifically, bio-transformation of human waste and CO_2_ would enable the production of food/water/O_2_ to allow long-term human missions to the Moon and Mars [3,7,83,84]. Figure 4 graphically shows the MELiSSA BLSS loop concept (Walker and Granjou, 2017). In recent years, members of the MELiSSA project have proposed numerous approaches for the biological valorisation of human urine.

Biological ammonification or nitrification was often chosen as a way to break down organic nitrogen compounds, e.g., urea and amino acids into NH_3_ or, preferably, NO_3_^−^. That is because it is easier to monitor and control the load and uptake of nitrogen when it is in its inorganic form. Nitrate, specifically, is preferred as it is more stable and less toxic compared to NH_3_. In fact, NH_3_ is alkaline, volatile and toxic at high concentrations [3]. While several articles have shown that aerobic biological nitrification of urine is possible [49,85,86], until 2018 it was unclear whether the microbial consortium could survive the level of radiation and gravity on the ISS. However, in 2018, a research team sent a nitrifying microbial consortium on a 44-day FOTON-M4 flight to Low Earth Orbit (LEO) and exposed it to 10^−3^–10^−4^ g (gravitational constant) and 687 ± 170 µGy (Gray) d^−1^ (20 ± 4 °C), exposing them to about double the radiation level of that on the ISS [87]. Interestingly, upon reactivation, the consortium was able to perform ureolysis, ammonia and nitrite oxidation at the same rate as before. Additionally, the bottleneck of providing fine bubble aeration in the absence of gravity could be overcome by using flat sheet or hollow fibres membrane aeration systems [88]. This opens up the possibility of converting human urine into protein-rich algae biomass [89]. Finally, more research should be conducted on tackling the issue of accumulation of recalcitrant micropollutants in the bioreactor. To cope with this problem, new solutions should also target either the selective separation between nutrients and micropollutants, or the destruction of the pollutants via advanced oxidation.

### 5.2. Water Wall Concept

**Figure 5:**
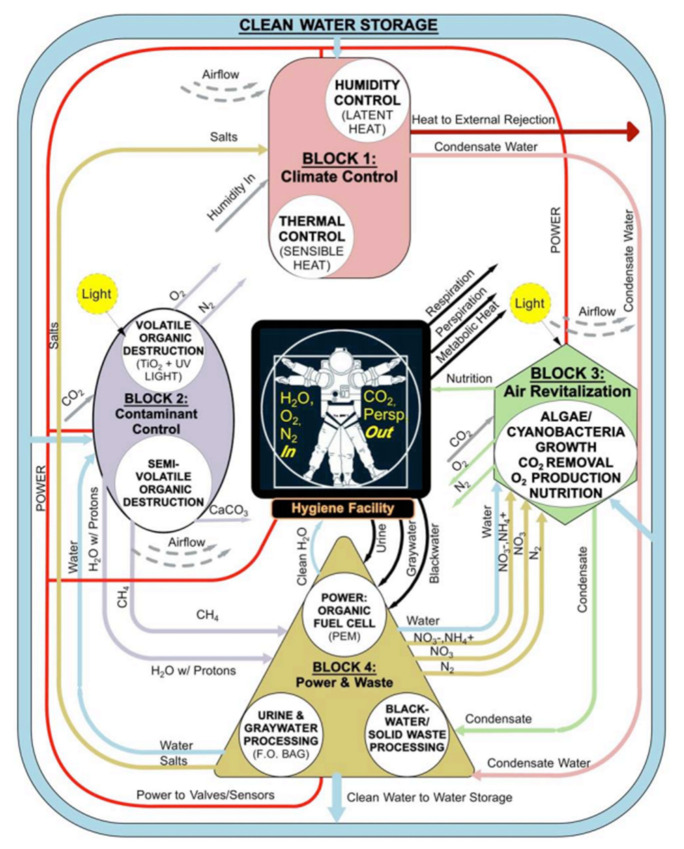
Initial concept of water wall system architecture. Credit to Cohen et al., 2014. Permission granted by the copyright holder (Marc M. Cohen).

NASA also developed a similar closed-loop concept. Their design, however, focused also on the reuse of concentrated wet wastes as a way of shielding the crew from cosmic radiation [2,90]. Their idea was to create a water wall comprised of a series of FO membrane bags packed as dry elements integrated into an inflatable habitat structure’s wall. After launch into orbit, the FO bags are filled with produced wastewater to extract potable water from it. Once the FO bags are exhausted or fouled, they are filled with faeces, solid organic wastes and other wastewater brine residuals. The bags now operate as anaerobic digestion bags, where the produced CH_4_ and CO_2_ are harvested for O_2_ generation. After digestion, the stabilised solids are combined with urine brine and become a permanent hydrocarbon/hydrated precipitate radiation shield [2]. Figure 5 graphically shows the water wall concept.

Reviewing the available technologies for nitrogen cycling in the BLSS, Clauwaert et al. (2017) [3] concluded that most of the scientific research focused on the recovery of water, oxygen and carbon, while other (micro) nutrients are often neglected. However, efficient management of nutrients is essential to guarantee quasi-independent, extra-terrestrial colonisation, as the resupply for an increasing population would not be feasible.

## 6. Conclusions

The fundamental question of sourcing the adequate and necessary supply of water and food to the crew has to be addressed to allow for life support during long-term space missions, and to achieve this, urine treatment and reuse as drinking water and nutrient solution will be of paramount importance. While advances have been made in improving the treatment and reuse of water from the crew’s urine and latent water, this review has identified several aspects that still have to be improved. Among the necessary upgrades to the UPA and WPA, the following were identified as especially significant:

➢ broadening the types of wastewaters that the WRM can process and increasing the current recovery rates beyond 85%,
➢ improving the resilience of the system by tackling the critical issue of “dormancy”, defined as the concerns of microbial growth or chemical degradation that would prevent water systems from operating once crew returned to the vehicle,
➢ improving the reliability, redundancy and weight of the system while minimising the use of consumables,
➢ investigating the use of “green pre-treatments” of urine to allow for the subsequent reuse of wastewater brine as a nutrient solution.

Finally, only by reducing the use of expendable materials and minimising the need for tools and spare parts to replace broken components can human-crewed, long-distance missions be truly attempted. To conclude, transforming the produced wastes (e.g., brine from the UPA and WPA) into usable and valuable products will have to be increasingly investigated to address both the waste disposal concern and the access to nutrients and raw materials necessary for higher plant production or radiation protection. New processes capable of closing the nutrients loop are increasingly needed so survive the 30-month mission duration.

## Figures and Tables

**Table 1:** Organic and inorganic chemical composition of human urine. The results from the first two rows are from the analysis conducted at the University of Technology Sydney (UTS) urine source separation system. The main difference between waterless urinals and urine-diverting toilets is that, in the latter, urine gets diluted two to four times with tap water.

	EC	COD	Urea	TAN	Creatinine	PO_4_^3−^-P	K^+^	Mg^2+^	Na^+^	Ca^2+^	B	Mn	Fe	Cu	Zn
	[mS/m]	[mg/L]	[g/L]	[mg/L]							[µg/L]				
UTS Waterless Urinal (hydrolysed urine) ^1^	40.5 ± 0.7	6174 ± 621	n.d.	6817 ± 145	-	178 ± 15	1604 ± 112	13 ± 21	1971 ± 129	5 ± 31	883 ± 101	<1	19 ± 8	6.7 ± 1.1	200 ± 34
UTS Urine Diverting Toilet (hydrolysed urine) ^1^	25.9 ± 0.4	4873 ± 728	n.d.	3846 ± 121	-	85 ± 5	1387 ± 97	36 ± 25	1204 ± 83	7 ± 20	1170 ± 50	<1	23 ± 11	18 ± 3	137 ± 31
Fresh Urine ^2^	15.5–19.6	-	9.3–23.3	200–730	670–2150	470–1070	750–2610	102–205	1170–4390	30–390	435–440	0.062	165–205	13 ±11	19–665
Urine Collected from the Crew of “Lunar Palace 1” ^3^	-	-	25,745 ± 1535	304 ± 4	1300–1500	1186 ± 3	1629 ± 53	146 ± 10	8740 ± 81	110 ± 8	-	-	-	-	-

^1^ [25,26], ^2^ [11,27,28], ^3^ [6].

**Table 2:** Pharmaceutical and hormone concentrations in human urine from the literature. The results from the first two rows are from the analysis conducted at the University of Technology Sydney (UTS) urine source separation system. The main difference between waterless urinals and urine-diverting toilets is that, in the latter, urine gets diluted two to four times with tap water.

	Caffeine	Carbamazepine	Ibuprofen	Naproxen	Estrone	Estriol	Fluoxetine	4-Acetamidophenol	Triclosan	Diclofenac
UTS Waterless Urinals ^1^ [µg/L]	1475 ± 35	37 ± 3	497 ± 41	<10	<10	6 ± 4	12 ± 2	477 ± 278	48 ± 9	<10
UTS Urine Diverting Toilet ^1^ [µg/L]	2165 ± 145	<5	<10	197 ± 195	<10	9 ± 7	<5	2700 ± 1560	25 ± 4	<10
	**Diclofenac**	**Sulfamethoxazole**	**N4 Acetyl-SMX**	**Trimethoprim**	**Hydrochlorothiazide**	**Atenolol Acid**	**Ritonavir**	**Atenolol**	**Emtricitabine**	**Clarithromycin**
Concentration Range ^2^ [µg/L]	3.2–72	<2–6800	<1–3500	<2–1300	<3–134	<4–1100	<1–4.6	<1–300	<6–920	<1
Frequency of Occurrence ^2^	100%	95%	90%	85%	80%	75%	70%	55%	40%	20%

^1^ [25,26], ^2^ [24].
